# Pattern of Oculomotor Findings in Children with Attention Deficit/Hyperactivity Disorder in Relation to Methylphenidate Treatment

**DOI:** 10.3390/jcm15062108

**Published:** 2026-03-10

**Authors:** Claudia Brogna, Valentina Napoli, Laura Castellini, Federica Mirra, Simona Sestito, Giuseppe Marsella, Maria Luisa Piscopiello, Valentina Cima, Daniela Ricci, Annabella Salerni, Gianluigi Di Cesare, Patrizia Brogna, Domenico M. Romeo

**Affiliations:** 1 Pediatric Neurology Unit, Università Cattolica del Sacro Cuore, 00168 Rome, Italygiuseppe.mars3@gmail.com (G.M.); domenicomarco.romeo@policlinicogemelli.it (D.M.R.); 2 Pediatric Neurology Unit, Fondazione Policlinico Universitario A. Gemelli IRCCS, 00168 Rome, Italy; 3 Ophthalmology Unit, Fondazione Policlinico Universitario A. Gemelli IRCCS, 00168 Rome, Italy; valentina.cima@policlinicogemelli.it (V.C.);; 4 National Centre of Services and Research for the Prevention of Blindness and Rehabilitation of Visually Impaired, IAPB Italia Onlus, 00168 Rome, Italy; 5 Prevention and Early Intervention Complex Operative Unit, Department of Mental Health, ASL Roma 1, 00193 Rome, Italypatrizia.brogna@aslroma1.it (P.B.)

**Keywords:** oculomotor alterations, ADHD, methylphenidate

## Abstract

Attention-Deficit/Hyperactivity Disorder (ADHD) may be associated with alterations in eye movements, which in turn may reflect dysfunctions in executive functions and sensorimotor integration processes. This review analyzed the pattern of oculomotor findings of pediatric populations diagnosed with ADHD with or without methylphenidate (MPH) treatment, with the aim of systematically describing the oculomotor abnormalities observed in affected children. A total of 24 studies were analyzed. The results showed that children with ADHD exhibit increased latency, a higher number of directional errors in prosaccade and antisaccade tasks, as well as intrusions during fixation and a higher frequency of microsaccades and involuntary blinks. Furthermore, studies involving the administration of MPH showed an improvement in oculomotor control, with a reduction in errors and a modulation of latency and oculomotor inhibition. These findings confirmed the potential role of oculomotor parameters as objective and non-invasive biomarkers for exploring the neurofunctional correlates of ADHD and for evaluating the effects of pharmacological treatment.

## 1. Introduction

In the last few years, there has been increasing attention on the implications of oculomotor alterations in learning and cognitive functions in both typically developing children and in those with neurodevelopmental disorders [1,2]. This interest stems both from the rising prevalence of such disorders during developmental age and from the opportunity to non-invasively investigate the functioning of the neurophysiological circuits involved. Indeed, oculomotor activity represents a fine motor function that can be easily measured in children using simple, non-invasive tasks and procedures [3,4]. Karatekin et al. hypothesized a potential involvement of the oculomotor pathways in neurodevelopmental disorders [5,6] as the oculomotor control involves the integration of complex brain structures, including the cerebellum, superior colliculus, basal ganglia, thalamus, and cerebral cortex [7]. In recent years, interest in Attention-Deficit/Hyperactivity Disorder (ADHD) has grown significantly, particularly with regard to its clinical manifestations, neurobiological underpinnings, and functional implications for cognitive and behavioral development in children. ADHD is a neurodevelopmental disorder with onset before the age of 12 years that impairs adaptive functioning or development in at least two settings (e.g., home, school, social environments). The global prevalence of ADHD in school-aged children is estimated at approximately 5% [8,9]. ADHD has a multifactorial etiology, involving complex interactions between genetic and environmental factors [10,11]. During the school years, the disorder significantly affects health-related quality of life, impairing academic performance and psychosocial well-being [12]. A substantial proportion of children with ADHD also meet diagnostic criteria for specific learning disorders (SLDs), such as dyslexia or dyscalculia [9,13]. Individuals with ADHD frequently show executive function impairments, difficulties in performing complex tasks, attentional instability, and slower processing speed [14,15]. Neurofunctional alterations documented through neuroimaging studies include reduced activation of the anterior frontal cortex and basal ganglia [16], as well as decreased volume in sensorimotor, frontal, and cerebellar regions [17]. Furthermore, atypical development of connections between fronto-striatal and cerebellar regions has been proposed as a possible mechanism underlying the motor impairments observed in patients with ADHD [14].

Among the various manifestations associated with ADHD, in recent years, growing interest has emerged regarding alterations in oculomotor control mechanisms, which serve as important indicators of executive functioning and sensorimotor integration [18,19,20,21,22,23], both in typically developing children and in those with neurodevelopmental disorders [1,2]. Oculomotor alterations may significantly affect children’s academic functioning, contributing to difficulties in reading and writing [24]. In patients with ADHD, numerous studies employing eye-tracking technologies reported correlations between abnormal eye movements and executive or attentional dysfunctions [14,25]. Children with ADHD have been observed to exhibit a higher frequency of blinking and microsaccades during the peri-stimulus period, a phase during which such movements should typically be inhibited to allow accurate visual processing.

In a recent meta-analysis, Chamorro et al. [19] reported an interesting topic on the role of the oculomotor inhibition in adults and children with ADHD, suggesting that oculomotor disinhibition and, more specifically, saccadic reaction time variability, are relevant ADHD biomarkers.

Children with ADHD are differentiated from adult ones due to the different cerebral maturation network involved. These differences between children and adult populations have been addressed in two recent meta-analyses that confirm age-related changes in brain activations related to ADHD, though somehow differing in the loci and nature of these changes. While one found hypoactivation differences only [20], the other found hypo- and hyperactivation changes between children and adults with ADHD [21], confirmed also by other studies [22,23].

By critically reviewing the existing literature, the present study aims to highlight the clinical relevance of oculomotor abnormalities in children with ADHD, providing a detailed overview of typical alterations and changes observed following methylphenidate (MPH) administration. Specifically, this review includes studies that have investigated task-based assessments of several oculomotor functions, such as saccadic movements, ocular fixation (including intrusive movements), microsaccades, the frequency of involuntary blinking, and, to a lesser extent, smooth pursuit eye movements. More specifically, saccadic movements included: (i) prosaccades, which are rapid, automatic eye movements toward a visual stimulus; (ii) antisaccades, which require suppression of a reflexive glance toward a peripheral stimulus and generation of a voluntary saccade in the opposite direction; and (iii) memory-guided saccades, which assess the ability to maintain the spatial location of a target in working memory and direct gaze toward it after a delay when the target is no longer visible.

## 2. Materials and Methods

### 2.1. Search Criteria

**Figure 1:**
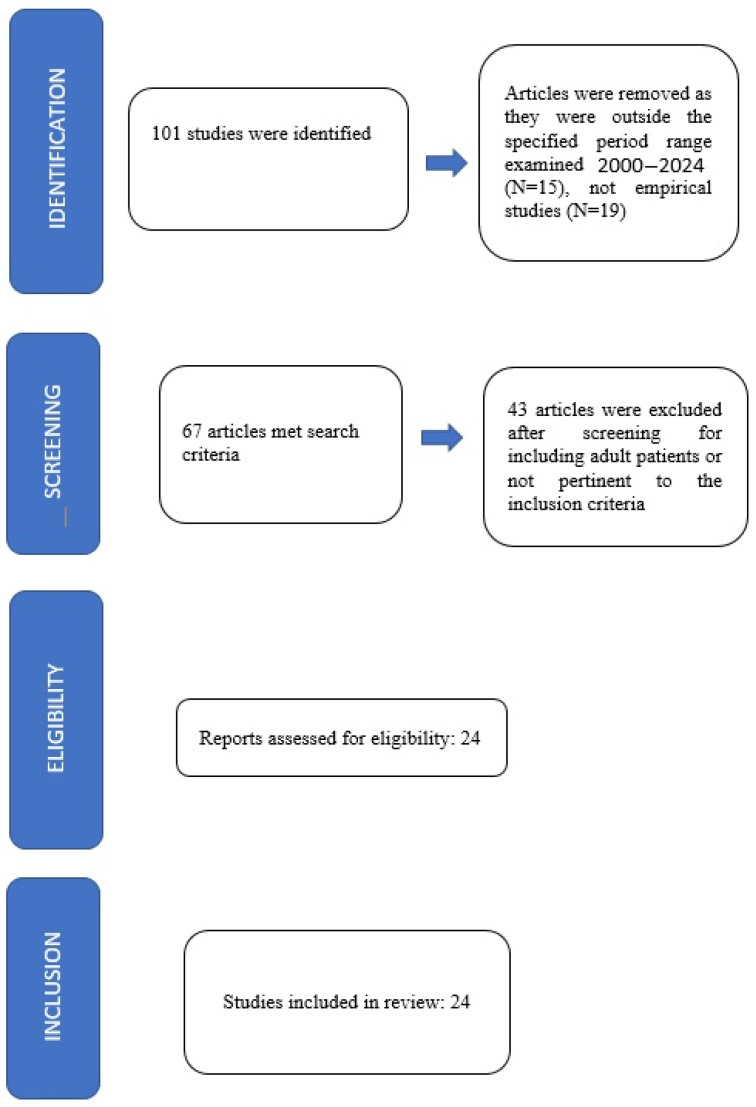
Flow chart for process according to PRISMA.

The review was carried out according to the guidelines Preferred Reporting Items for Systematic Reviews and Meta-Analyses (PRISMA; see Figure 1).

The research was conducted using the following databases: PUBMED, MEDLINE, Embase, and Scopus.

Search terms used were “Attention-Deficit Hyperactivity Disorder”, “oculomotor”, “eye movements” and “methylphenidate”.

### 2.2. Inclusion Criteria

Studies were selected for inclusion if they were written in English or if a suitable English translation was available, and if they were human-based. All the studies were first selected, looking for the presence of a clinical association between “ADHD”, “oculomotor”, “eye movements” and “methylphenidate”. Studies published within the interval range 2000 to 2024 were included. We included studies with an age range of 0–18 years.

### 2.3. Exclusion Criteria

The exclusion criteria for this review were clearly defined before the screening process began. Specifically, in the course of identifying relevant studies, articles were excluded if they were case reports or analyzed adult subjects.

### 2.4. Data Extraction and Analysis

The title and abstracts of the studies were independently examined for suitability by four authors (V.N., L.C., F.M., M.L.P.) and critically checked by a third independent reviewer (C.B.); conflicting viewpoints were discussed until a consensus was reached. A total of 101 studies were initially identified; 15 were excluded because they fell outside the predefined year range, while 19 were removed as they did not meet the established eligibility criteria for empirical research (i.e., reviews, systematic reviews, meta-analyses, etc.); after a review of the full text, 43 were excluded, as they included adult samples or were not pertinent to the inclusion criteria. The remaining 24 articles were included in the present review (details are reported in Table 1) [24,25,26,27,28,29,30,31,32,33,34,35,36,37,38,39,40,41,42,43,44,45,46,47].

## 3. Results

In this chapter, we analyzed the findings emerging from the literature, organizing them into subsections dedicated to several aspects of oculomotor findings, including (i) saccades, (ii) fixation, and (iii) smooth pursuit, highlighting the main alterations observed in children with ADHD compared to typically developing peers [24,25,26,27,28,29,30,31,32,33,34,35,36,37,38,39,40,41,42,43,44,45,46,47].

The first section examines studies describing oculomotor abnormalities in the absence of pharmacological treatment (untreated ADHD subjects) [24,25,26,27,28,29,30,33,36,37,38,39,41,43,45,47], while the second one presents findings regarding the effects of stimulants, like MPH, on oculomotor function [26,27,28,30,31,32,34,35,40,42,44,46]. Visual tracking intervention or training of eye movements has not been reported in all the studies investigated. See Table 1 for details.

## 4. Oculomotor Findings in Untreated Young Subjects with ADHD

### 4.1. Saccades: Prosaccades (Reflexive Saccades), Antisaccades and Memory-Guided Saccades (MGSs)

Saccades represent the most extensively studied eye movements in children with ADHD, as they sensitively probe inhibitory control, motor preparation, and movement precision. Our review gathered multiple findings describing significant alterations across various experimental paradigms [26,27,28,29,30,31,32,33,34,35,36,37,38,39,40,41,42,43,44,45,46,47]. The saccadic movements analyzed include prosaccades, antisaccades, and memory-guided saccades. We report the related findings investigated in in Young subjects with ADHD without medication (medication naive) [24,25,26,27,28,29,30,33,36,37,38,39,41,43,45,47].

Prosaccades are rapid, automatic eye movements toward a visual stimulus. Several studies in children with ADHD report anomalies in latency and response variability [25,26,27,28,29,35,36,37,38,47]. For example, Mostofsky et al. [26] found an increased intra-subject latency variability despite comparable mean latency, suggesting an impaired motor preparation and sustained attention, implicating prefrontal mechanisms responsible for executive control of movement. Mahone et al. [27], examining a larger sample of 60 children with ADHD and 60 typically developing peers aged 8 to 12 years, further observed prolonged latency in the overlap paradigm—a task requiring inhibition of central fixation before initiating a peripheral saccade—indicating impaired motor preparation. This study [27] also noted a greater variability in prosaccadic latency and a significant sex effect, with females exhibiting longer latencies independent of symptom severity. These findings suggested that even automatic prosaccades may be affected by motor planning deficits modulated by individual factors such as sex. In a sample of 31 medication-naïve children with ADHD and 31 matched controls, Bucci et al. [28] reported shorter mean prosaccadic latencies in the ADHD group, specifically in the overlap paradigm, alongside increased saccades during fixation and more direction errors in antisaccade tasks.

Loe et al. [37] reported no differences in overall percent correct, latency, or accuracy of saccades in the ADHD group compared to controls, while in other studies, children with ADHD were slower than the controls in the latency and total search time of the saccade [36,38], but they were accurate in their eye movements like the controls [38]. This result is in contrast with other studies showing children with ADHD to be less accurate in their saccades [39] with no differences for latency [45]. When comparing with other neurodevelopmental disorders [29] in children with ADHD, the latency of the visual target was not affected by posture, compared to the control group.

The antisaccade task requires suppression of a reflexive glance toward a peripheral stimulus and generation of a voluntary saccade in the opposite direction. This paradigm is widely used to assess inhibitory control and is particularly sensitive to executive dysfunction. All included studies [25,26,29] report significantly increased direction errors in children with ADHD relative to typically developing peers. Mostofsky et al. [26] confirmed marked increases in antisaccade errors, interpreting them as manifestations of prefrontal dysfunction.

Similarly, Klein et al. [30] examined 46 children with ADHD and 46 controls, observing increased reaction times in both pro- and antisaccades, elevated direction errors, a higher rate of anticipatory responses, and reduced saccade frequency in the gap condition in children with ADHD. Supporting these findings, Bucci et al. [28], in their group of 31 unmedicated children with ADHD, also reported more direction errors, further emphasizing the difficulties in suppressing automatic oculomotor responses. Hanisch et al. [35], in a group of 22 children with ADHD and 22 healthy controls, identified selective oculomotor inhibition impairments, with children with ADHD struggling to inhibit prepotent responses, especially in novel or complex contexts, consistent with fronto-striatal inhibitory circuit dysfunction. Huang and Chan [31] also contributed to these findings with a smaller sample of 12 children with ADHD and 12 controls, reporting significantly higher error rates and shortened latencies in antisaccade tasks in the ADHD group. Similar results are reported by Karaketin et al., showing impaired antisaccade accuracy with more premature saccades and fewer corrective saccades [40], and by Loe et al. [37]. Fernandez-Ruiz et al. [32] reported data of the antisaccade task of 22 ADHD children compared with 20 controls while undergoing an fMRI study with concurrent eye tracking. The ADHD group showed longer reaction times and more antisaccade direction errors than controls and a significant hyperactivation in the dorsolateral prefrontal cortex.

MGS tasks assess the ability to maintain the spatial location of a target in working memory and direct gaze toward it after a delay when the target is no longer visible. This requires integration of visuospatial memory, motor preparation, and inhibitory control. In the study by Mostofsky et al. [26], the ADHD group reported significantly more anticipatory errors and longer latencies than controls, though spatial accuracy was preserved, suggesting difficulties in planning and executive control rather than spatial coding. Similar results are reported by other studies suggesting alterations in the functioning of the cerebellum, or alterations in the dopaminergic transmission, which would only affect memory-guided saccades [37,44]; Mahone et al. [27] validated these results, highlighting deficits in motor preparation (latency and variability) but not a generalized working memory impairment. However, inattentive-ADHD subtypes showed reduced accuracy in working memory tasks compared to combined subtypes, indicating subtype-specific differences. Damyanovich et al. [24] examined 41 ADHD children and 22 controls, describing difficulties in post-saccadic fixation, exacerbated during complex, multi-step tasks. These impairments involved challenges in maintaining fixation after saccades, particularly during demanding sequences, and may resemble the demands of MGS tasks, although MGSs were not directly tested. When comparing with other neurodevelopmental disorders [29] in ADHD children, the percentage of errors in MGSs was affected by postural condition, and it was improved when the child was standing up on the platform compared to sitting comfortably on a chair.

### 4.2. Fixation

Visual fixation is critical for maintaining stable gaze on a target and underpins visual exploration and attentional control. Children with ADHD exhibit fixation instability caused by intrusive saccades, inappropriate microsaccades, and difficulty sustaining gaze. Muñoz et al. [25] found in ADHD subjects an exogenous fixation control preserved but an endogenous fixation control impaired; these results suggested a reduced suppression of reflexive saccades, increased intrusive saccades during prolonged fixation, and greater variability in saccadic reaction times in ADHD patients. Gould et al. [33], in 53 children with ADHD and 44 controls, confirmed more large saccades (>4°) during simple fixation in the ADHD group, indicating a basic oculomotor control deficit even without complex cognitive demands. Notably, performance did not significantly improve during placebo testing, suggesting the persistence of underlying control impairments. In line with these findings, Bucci et al. [28] reported more saccades during fixation in medication-naïve children with ADHD, highlighting inhibitory control deficits in simple visual tasks. Damyanovich et al. [24], based on a clinical group of 41 ADHD children, identified post-saccadic fixation difficulties, worsening during complex tasks, potentially linked to immature frontal cortical function that may affect academic skills such as reading and writing. Bucci et al. [29] found that in the ADHD sample, the visual fixation was poor while the child was standing up on the platform, compared to the control group. Caldani et al. [43] showed that ADHD children had poor fixation capability and poor postural stability when compared to controls. Loe et al. found that increased interstimulus (IS) fixation periods (defined as the delay between the instructional cue and the appearance of the stimulus) on the antisaccade task was associated with improved performance and decreased reaction times on correct trials for controls but not for children with ADHD [37], whereas Matsu et al. found a longer reaction time and less stable eye-movement during the fixation period in ADHD children respect to the controls [47].

### 4.3. Smooth Pursuit

Smooth pursuit eye movements enable continuous tracking of a moving target, crucial for fluid visual processing. Compared to saccades and fixation, this domain is less investigated in ADHD children. Castellanos et al. [45] assessed smooth pursuit alongside delayed response and inhibitory control tasks in girls only with ADHD. They found no significant differences in pursuit performance compared to controls, suggesting relative preservation of this oculomotor function in ADHD. However, no specific data on participant number was reported in this section.

## 5. Oculomotor Findings in Young ADHD Subjects Treated with Stimulants

Despite the increasing use of methylphenidate (MPH) in the treatment of ADHD, studies specifically investigating its effect on the improvement of oculomotor movements remain limited in the literature [26,27,28,30,31,32,34,35,40,42,44,46]. MPH dosage, formulation, and wash-out periods are not always reported in the original manuscript. In our review, one article analyzed the changes in oculomotor parameters before and after the administration of MPH [46]. Only two studies [30,31] reported the formulation of the drug used, indicating the use of Ritalin and Concerta. Finally, in three studies, the dosage used was specified [26,28,42]. The selected studies were conducted on pediatric samples, using computerized oculomotor tasks administered pre- and post-treatment, or comparing treated versus untreated subjects. Overall, the results suggest a favorable effect of MPH on oculomotor control, with evident improvements particularly in tasks requiring voluntary inhibition and sustained visual attention. Specifically, we analyzed the changes observed in saccadic tasks, fixation, and smooth pursuit.

### 5.1. Saccades: Prosaccades (Reflexive Saccades), Antisaccades and Memory-Guided Saccades (MGSs)

Bucci et al. [28] examined 31 children with ADHD and 31 matched controls before and after one month of MPH treatment. Results showed normalization of voluntary saccade latency in the gap, step, and overlap paradigms, and a significant reduction in errors during the antisaccade task after medication. Oculomotor performance in the ADHD group became comparable to that of controls after medication. The performance of saccades (pro- as well as antisaccades) is similar in children with ADHD (off and on MPH) and controls [44].

Mostofsky et al. [26] included 19 children and adolescents with ADHD (11 unmedicated, 8 on MPH) and 25 typically developing controls. The group receiving MPH showed less variability in prosaccade latency and fewer anticipatory errors in MGSs, although MPH did not fully correct response inhibition deficits. Klein et al. [30] evaluated 46 children with ADHD (6 tested under MPH, 40 in wash-out) and 46 controls. Medicated children exhibited improved performance in pro- and antisaccade tasks, with reduced anticipatory errors and shorter reaction times. Notably, age-related improvements in latency were less evident in the ADHD group. Hanisch et al. [35] tested 22 children with ADHD and 22 controls, after a ≥12 h medication wash-out, showing that fixation difficulties in ADHD are not due to generalized motor impairment but to selective inhibitory dysfunction. Children with ADHD struggle to suppress automatic responses even in routine contexts, suggesting fronto-striatal inhibitory circuit abnormalities. Huang & Chan [31] studied 12 children with ADHD and 12 controls; participants had discontinued MPH use 24 h before testing. Despite the wash-out, ADHD children showed greater inaccuracy and more direction errors in antisaccade tasks, suggesting persistent oculomotor dysregulation, though possibly attenuated by treatment history. Fernandez-Ruiz et al. [32] did not report a drug-related activation difference, but they showed a significant correlation between the difference in OFF/ON preparatory activation in the precuneus and a decrease in the number of antisaccade errors. Schwarz et al. [34] studied the correlation between antisaccade performance and neural correlates on fMRI in 11 children with ADHD and children treated with MPH matched to 11 typically developing children; the authors reported a greater brain activation in regions of the right dorsolateral prefrontal cortex and caudate nucleus in ADHD children without any difference in antisaccade performance between the two groups. MPH significantly improved motor planning and response inhibition, evaluated with antisaccade, as also reported by Klein et al. [46]; MPH decreased saccade latency but did not affect saccade amplitude or peak velocity, decreasing antisaccade percent error and latency and error rates [42].

### 5.2. Fixation

Bucci et al. [28] also assessed fixation tasks before and after MPH treatment. Following one month of therapy, children with ADHD exhibited a drastic reduction in intrusive saccades during fixation, with post-treatment performance reaching levels observed in typically developing peers. In a previous work, Bucci et al. reported that children with ADHD (off and on MPH) made significantly more saccades during the fixation task than the control group [44].

### 5.3. Smooth Pursuit

Bucci et al. [44] reported no differences in pursuits in children with ADHD (off and on MPH) compared with controls.

## 6. Discussion

The aim of the present review was to analyze the existing literature on oculomotor findings in children with ADHD in response to growing scientific interest regarding the potential link between oculomotor alterations and neurofunctional ADHD cerebral dysfunctions [7,24,26,27,28,29]. The objective was to explore how different components of oculomotor control reflect specific abnormalities in neural circuits implicated in the regulation of executive and attentional functions in this population and how they can change with the use of stimulants. This is an important issue to investigate as oculomotor functions are involved in several aspects of daily life in children, including learning processes affecting school adaptive functioning, which in ADHD children are just compromised by poor attention and impaired impulsivity. These considerations support the importance of adopting early rehabilitative approaches that could address not only higher-order executive functions but also underlying oculomotor mechanisms.

Executive and attentional difficulties typical of ADHD emerge more clearly in oculomotor tasks requiring visuospatial working memory, motor preparation, and inhibition of automatic responses, such as antisaccades and memory-guided saccades [26,27]. Consistent with the literature, our analysis found that saccadic eye movements have been the most extensively investigated, with particular focus on prosaccades, antisaccades, and memory-guided saccades [26,27,28,29,30,31]. Prosaccades, while less altered, provided useful information on reaction times and motor response preparation [27,29].

These findings are consistent with previous systematic reviews and meta-analyses documenting robust oculomotor deficits in children and adolescents with ADHD, particularly in tasks requiring inhibitory control and executive regulation [48,49].

Regarding smooth pursuit eye movements, available data are currently limited. In particular, Castellanos et al. [45] reported that smooth pursuit was largely preserved, confirmed also by Bucci et al. [44] in a population of treated and untreated children with ADHD. However, although altered pursuit performance has been observed in ADHD, further research is needed to determine whether and how MPH modulates this oculomotor domain. This highlights a significant gap in the literature, warranting further investigation through targeted experimental studies.

To evaluate antisaccade movements, subjects are required to suppress the voluntary response to look at a peripheral target in order to look in the opposite direction. The antisaccade task has been confirmed as the most sensitive oculomotor measure for detecting inhibitory control deficits in children with ADHD. Notably, while reaction times to antisaccades decrease with age in typically developing children, this maturation effect is absent in ADHD, suggesting developmental anomalies in executive functions. These results aligned with Barkley’s neuropsychological model, positing prefrontal cortex dysfunction as the basis for inhibitory deficits [30]; indeed, several studies confirmed altered frontostriatal activation during the execution of this task, covering with the neural correlates of ADHD, supporting the role of the atypical maturation of fronto-striatal circuits involved in voluntary cognitive control [24,25,26,27,28,29,30,31]. So far, in typically developing children, only a few fMRI studies have examined the neural correlates of antisaccade performance [50,51], showing a basic saccadic circuitry less well integrated compared with adults. Several studies reported in children a greater brain activation in the dorsal anterior cingulate cortex and a reduced activation in the right dorsolateral prefrontal cortex (dlPFC) compared to adults [50,51]. There are few data only of fMRI studies focusing on an antisaccade paradigm in children with ADHD showing significantly greater brain activation in regions in right dorsolateral prefrontal cortex and caudate nucleus in children with ADHD relative to the control group [19,34]. Antisaccade performance did not significantly differ between the groups, perhaps as a result of greater brain activation or medication effects in the ADHD group [34].

Alterations in eye movements provide a clinically objective method to investigate the neurofunctional mechanisms underlying the executive and attentional deficits characteristic of this disorder. From a clinical perspective, the analysis of eye movements is essential for the diagnosis and definition of neurocognitive profiles, but also to guide rehabilitative therapy and monitor the response to pharmacological treatments; mainly, the efficacy of methylphenidate on the inhibitory mechanisms of the oculomotor system as measured with the antisaccade task should be underlined [28,42].

A clinically relevant aspect emerging from this review is the use of oculomotor assessments not only for diagnostic purposes but also as objective, non-invasive, and repeatable tools to characterize pharmacological treatment response. The effect of methylphenidate (MPH) is clearly evident in some studies, but not in others; despite the increasing use of MPH in the treatment of ADHD, studies specifically investigating its effect on the improvement of oculomotor movements remain limited in the literature. Some studies only [26,27,28,30,31] analyzed oculomotor performance before and after methylphenidate administration, demonstrating significant beneficial effects of MPH on oculomotor control, with evident improvements particularly in tasks requiring voluntary inhibition and sustained visual attention [26,27,28,29,30,31,32,33]. Specifically, improvements were observed in latency times, reductions in directional errors, and increased latency in voluntary eye movements, interpreted as an index of improved inhibitory control [26,28]. The MPH effect is probably related to the direction of the drug on the same cerebral areas involved in saccadic movements. Indeed, even if there are contrasting results revealing no significant activation change in the dlPFC [34], FMRI investigations using placebo-controlled stimulant medication designs have shown normalized or greater brain activation in children and youths with ADHD who received MPH compared to control groups in regions associated with antisaccade performance, particularly right dlPFC, IFG, and striatum [34].

Previous studies reported on an increased brain activation in the dorsolateral prefrontal cortex (dlPFC), inferior frontal gyrus (IFG), and striatum in children and adolescents with ADHD who received stimulant medication such as MPH than control groups [52,53,54]. This effect is probably due to a modulating dopamine transmission, resulting in increased extracellular dopamine and amplified cortico-striatal signal [55,56], leading to enhanced motivation and interest to engage in tasks, increase attention and decrease distractibility, all of which improve CC-related functions.

In children with ADHD, a decrease in functional connectivity between the precuneus and anterior cingulate and ventromedial prefrontal cortex was also reported [50]. These results are also supported by previous findings in performance, monitoring and vigilant attention, suggesting a possible MPH effect on a system including the precuneus [53,54]. On the other hand, the lack of MPH effect on oculomotor findings reported in other studies is probably related to the presence of other comorbidities that have not always been reported or to several formulations used, also not always reported. In particular, early experimental studies demonstrated that methylphenidate modulates saccadic performance by reducing error rates and improving response inhibition, especially during antisaccade tasks [46]. DeVito et al. reported that MPH improves response inhibition but not reflection-impulsivity in children with ADHD, providing additional evidence on the selective effects of stimulant medication on oculomotor and cognitive control functions [57].

From a clinical and rehabilitative perspective, the findings summarized in this review suggest that specific oculomotor tasks—particularly antisaccades and memory-guided saccades—may represent sensitive functional markers of executive and inhibitory control deficits in children and adolescents with ADHD. The partial normalization of oculomotor performance following methylphenidate administration supports the potential role of eye movement assessment as a complementary outcome measure for treatment monitoring. Repeated, non-invasive oculomotor evaluations may contribute to longitudinal assessment, allowing clinicians to better characterize individual neurofunctional profiles and to tailor pharmacological and rehabilitative interventions. Moreover, given the involvement of oculomotor functions in learning-related activities such as reading and visuospatial processing, these measures may also be relevant for the planning of targeted rehabilitation programs addressing both attentional and academic difficulties.

It should be noted that this review did not aim to provide an exhaustive synthesis of all available studies, but rather to discuss selected evidence relevant to the clinical implications of oculomotor findings in pediatric ADHD.

## 7. Limitations of the Study

There are several limitations that should be recognized. It is important to acknowledge certain methodological issues characterizing the available literature. First, there is a considerable heterogeneity in experimental protocols, with variations in recording methodologies (eye-tracking vs. electrooculography [EOG] and electroencephalography [EEG]) [24], in wash-out duration (ranging from 12 h to more than 10 days) [28,32], and in sample sizes, which are often small [26,31,32,33,34,35,36,37,38,39,40]. Furthermore, the majority of studies predominantly included male subjects, with few analyses addressing sex differences. Girls with ADHD are therefore underrepresented in the current literature, with the exception of Castellanos et al. [45], which exclusively examined female subjects. That study reported longer visual latencies in the VGS task compared to controls, suggesting possible sex-related differences in oculomotor mechanisms [27,33]. Lastly, the wide age ranges considered across studies (e.g., 6–16 years in Munoz et al. [25]) may have introduced additional variability related to neurocognitive development, which was not always adequately controlled for.

## 8. Conclusions

In recent years, several research articles have focused on the role of oculomotor abnormalities in ADHD subjects [19,57]. The assessment of eye movement in children and adolescents with ADHD offers important support in optimizing rehabilitative interventions; this is important to provide potential benefits for improving comorbidities frequently associated with ADHD, such as specific learning disorders, especially dyslexia, also due to the non-invasive, objective, and repeatable nature of these measurements.

In particular, oculomotor tasks involving voluntary inhibition and attentional control appear to be sensitive to the effects of methylphenidate, highlighting their potential utility in evaluating pharmacological efficacy at an individual level. Accumulating evidence, including recent systematic reviews and meta-analyses, supports the presence of oculomotor abnormalities as potential neuro-functional biomarkers in pediatric patients with ADHD.

For these reasons, it is crucial to promote further research focused on eye movement in children with ADHD, aiming to enhance methodological rigor, increase sample sizes including female participants, and deepen the understanding of clinical variables as well as the effect of stimulants. In this way, eye movement may eventually be established as a neurocognitive biomarker, improving clinical management and fostering increasingly personalized and effective interventions.

Future research should aim to improve methodological consistency, increase sample sizes with greater representation of female participants, and further clarify the specific effects of stimulant medications, including the type of stimulants and the dosage, on different components of oculomotor control. Additionally, emerging factors such as atypical sensory processing and increased digital media exposure, including smartphone use, should be considered, as they may influence oculomotor functioning in children with ADHD [48,58,59]. Advancing research in this area may contribute to establishing eye movement assessment as a reliable neurocognitive biomarker, ultimately improving personalized clinical management and rehabilitative outcomes.

Finally, further studies are needed to investigate the relation with MPH on several aspects of oculomotor findings in children, considering also atypical sensory processing that is common in ADHD subjects and influenced by smartphone use.

## Figures and Tables

**Table 1:** Oculomotor findings in young medication-naïve ADHD subjects and in young ADHD subjects treated with methylphenidate or other not specified stimulants. Note that MPH dosage, formulation, and wash-out periods are not always reported in the original manuscript.

Study	Design	Sample Size	Age (Years)	Oculomotor Test Used	Oculomotor Tasks	Main Findings	MPH or Other Medication Used	Limitations/Bias	Comorbidities
**Damyanovich et al. 2013 [24]**	Case–control	41 ADHD vs. 22 controls	7–9	EOG + EEG	Simple and coordinated tasks	ADHD: difficulty in post-saccadic fixation; greater instability in complex tasks.	Not reported	Unbalanced samples; indirect measurements	Not reported
**Munoz et al. 2003 [25]**	Case–control	76 ADHD vs. 75 controls	6–16	Eye tracking	Fixation, prosaccades, antisaccades	ADHD: impaired endogenous fixation control, more intrusive saccades, higher latency variability.	No: MPH, ciclosporin (no specified dose and formulation) were not used during the test, (minimal time between the previous dose and testing ≥ 20 h).	Wide age range; treatment inconsistently reported	Comorbidities excluded: learning disabilities, Tourette’s syndrome, bipolar disease
**Mostofsky et al. 2001 [26]**	Preliminary	19 ADHD vs. 25 controls	7–16	Electrooculography (EOG)	Prosaccades, antisaccades, memory-guided saccades	ADHD: more directional errors (antisaccades), more anticipatory errors (memory-guided), greater latency variability.	Yes: 8 ADHD children on MPH (dose 0.14–0.79 mg/kg per dose, taken 3 h to immediately before testing); 1 child also on imipramine (0.35 mg/kg/day). MPH improved consistency (reduced variability) and latency but did not significantly improve inhibition (antisaccade/anticipatory errors).	Small sample, cross-sectional, no within-subject ON/OFF design; mixed subtypes; stimulant dose/formulation not standardized.	Not reported
**Mahone et al. 2009 [27]**	Cross-sectional	60 ADHD (24 female) vs. 60 controls (29 female)	8–12	Cognitive oculomotor paradigms	Visually guided saccades (VGS), antisaccades, go/no-go, memory-guided	ADHD: slower and more variable VGS, impaired response inhibition, reduced memory in inattentive subtype. Girls with ADHD showed significantly longer VGS latency than ADHD boys and controls.	Yes: stimulant use allowed (type and formulation not specified); children taking stimulants withheld medication on the day of and the day before testing. No other psychoactive drugs permitted. Number of treated ADHD participants not reported.	Fixed task order; absence of ON/OFF medication comparison; stimulant regimen not standardized; no randomization of paradigms.	ODD (25–28%) and specific phobias (17–28%) allowed; other comorbidities excluded.
**Bucci et al. 2017 [28]**	Case–control	31 ADHD vs. 31 controls	~10	Eye tracking	Fixation, gap, step, overlap, antisaccades	ADHD: more saccades during fixation, more antisaccade errors, shorter latency (overlap). MPH: normalized parameters after 1 month.	Yes: 27 ADHD children treated with MPH (mean dose = 0.62 ± 0.04 mg/kg/day; formulation not specified); effects evaluated after 1 month of treatment.	Small sample; possible learning effect; absence of placebo group; single dose range (no dose–response analysis)	Comorbidities screened and excluded
**Bucci et al. 2018 [29]**	Case–control	26 ADHD vs. 26 controls	~9	Eye tracking	Saccades, memory-guided saccades, fixation according to postural condition	ADHD: the fixation task only had poorer eye-movement performances in the standing condition compared to the sitting condition.	Not reported	Heterogeneous clinical groups; no specific focus on ADHD-only subgroup; lack of pharmacological control.	Group 1 to 4 included, respectively, children with ADHD, children with dyslexia, children with high functioning (ASD) and typically developing children (TD).
**Klein et al. 2003 [30]**	Case–control	46 ADHD (5 female) vs. 46 controls (8 female)	9–13	Eye tracking	Pro- and antisaccades (gap and overlap)	ADHD: more anticipatory errors, longer reaction times, absent age-related latency decline.	Yes: 40 ADHD children were off MPH (Ritalin) for 12–18 h before testing; 6 received Ritalin 4–8 h prior to testing. Dosage not reported.	Sex imbalance groups; inconsistent drug suspension period.	Not reported
**Huang & Chan, 2020 [31]**	Case–control	12 ADHD (5 females) vs. 12 controls (4 females)	~9	View Hi-Speed (500 Hz)	VGS, antisaccades	ADHD: greater inaccuracy, more direction errors, shorter latency for distant targets.	Yes: children taking MPH (Ritalin or Concerta) discontinued medication 24 h prior to testing.	Small sample; non-uniform wash-out	Comorbidities explicitly excluded
**Fernandez-Ruiz et al. 2020 [32]**	Case–control	22 ADHD (5 females) vs. 20 controls (6 females)	~12.6	Eye tracking, fMRI	Antisaccades	ADHD: longer reaction times, more antisaccade direction errors, activations in saccade network areas, significant hyperactivation in the dorsolateral prefrontal cortex.	In the OFF meds condition, ADHD participants were asked to abstain from taking stimulant medication for at least 20 h before the MRI session; ADHD group analysis of OFF and ON stimulant medication did not show drug-related activation differences. Yes: (MPH, Atomoxetine): significant correlation between the difference in OFF/ON preparatory activation in the precuneus.	MRI acquisition, small sample size	Comorbidities excluded
**Gould et al. 2001 [33]**	Case–control	53 ADHD (29 females) vs. 44 controls (18 females)	7–13	Eye-tracker (Ober2)	21 s fixation	ADHD: more latency with large saccades (>4°) during simple fixation.	No: participants were medication-free for ≥10 days before testing; 23 females retested 3–9 weeks later under double-blind placebo. No medication dose or formulation reported.	Possible floor effect; weak correlations	Not reported
**Schwarz et al. 2015 [34]**	Case–control	11 ADHD (2 females) vs. 11 controls (3 females)	8–11	Antisaccade tasks, fMRI	Antisaccades	ADHD: significantly greater brain activation in regions in right dorsolateral prefrontal cortex and caudate nucleus was observed; no difference in antisaccade task.	Yes, children with ADHD were taking MPH; medication details and wash-out period not reported.	Small pediatric sample; lack of detailed medication information.	Not reported
**Hanisch et al. 2006 [35]**	Case–control	22 ADHD (7 females) vs. 22 controls (8 females)	~11–12	Eye tracking	Prosaccades, antisaccades, fixation	ADHD: similar saccadic response preparation and saccadic accuracy compared to controls, impaired antisaccades, fixation.	Yes: 13 patients on MPH, withheld ≥ 12 h before testing.	Fixed task order; small sample; limited randomization; exclusion of dyslexia and major comorbidities limits generalizability.	No comorbidities reported; dyslexia and other psychiatric disorders explicitly excluded.
**Goto et al. 2010 [36]**	Case–control	19 ADHD vs. 30 controls	6–11	Electrooculography	Saccade latency and accuracy, memory-guided saccade task measured with percentage of anticipatory errors (PAE), antisaccade task measured with percentage of direction errors (PDE)	ADHD: significant differences in saccade latency and accuracy, PAE and PDE rates compared to controls.	No: All subjects were free of medication for 24 h before the testing session. Formulation and dosage not specified.	Not reported	Not reported
**Loe et al. 2009 [37]**	Cross-sectional study	26 ADHD vs. 33 controls	8–13	Oculomotor task	Visually guided saccades, antisaccades, memory-guided saccades (MGSs), fixation	ADHD: no differences in latency, accuracy of saccades, but impaired antisaccades and MGSs and fixation than controls.	No: stimulant medication allowed but withheld on day of testing.	Small sample size, younger males and few females. ODD not excluded; some data only from parent vs. teacher discordant ratings.	4 ADHD-C and 5 ADHD-I had oppositional defiant disorder; learning problems excluded.
**Van Der Stigchel et al. 2007 [38]**	Case– control	22 ADHD, 20 controls	7–14	Oculomotor capture task	Prosaccades	ADHD had slower responses than controls, but were no less accurate.	No: psychostimulants discontinued ≥ 48 h before testing.	Small sample size; only male participants; absence of working memory measures	Not reported
**Rommelse et al. 2008 [39]**	Case–controls	14 ADHD, 15 controls	7–14	Oculomotor capture task and a memory-guided saccade task	Saccades, memory-guided saccades (MGSs)	ADHD: less accurate saccades, no differences for latency, alteration of MGSs compared to controls.	No: psychostimulants discontinued ≥48 h before testing.	Groups differed in IQ; small sample size; no female participants; reduced measures of working memory and inhibition.	Not reported
**Karatekin, 2006 [40]**	Case–control	10 ADHD (2 females) vs. 15 controls (6 females)	14–15	Eye tracking	Antisaccade accuracy and response time (RT)	ADHD: impaired accuracy and saccadic RT compared to controls.	Yes: 9 patients on psychostimulants (not specified), withheld 24 h before testing.	Small sample size, few number of trials in each condition	Oppositional Defiant Disorder (3), Conduct Disorder (1), Learning Disorder (2), Anxiety Disorder (3), Mood Disorder (2), Substance/Alcohol Use (2), Enuresis (1), Possible Sleep Disorder (1).
**Jostrup et al. 2024 [41]**	Case–control	52 ADHD (31 boys, 21 girls) vs. 45 controls (20 boys, 25 girls)	~11.7	Eye tracking, auditory white noise	Saccades, fixation, memory-guided saccades (MGSs)	ADHD: white noise stimulation does not appear to be beneficial for children with ADHD in tasks that target oculomotor inhibitory control.	No: MPH withdrawal ≥ 24 h before testing.	small sample; subtypes of ADHD, gender comorbidities, MPH discontinuation not physically inspected	Not reported
**O’Driscoll et al. 2005 [42]**	Case–control	22 ADHD vs. 10 controls	11.5–14	Eye tracking	Saccades, antisaccades	ADHD: no impairment on n visually guided saccades, elevated antisaccade percent error, significant main effect of Switch on percent error.	Yes: MPH administered (0.5 mg/kg to a maximum of 30 mg) formulation not specified. MPH significantly improved motor planning and response inhibition; MPH decreased saccade latency but did not affect saccade amplitude or peak velocity, decreased antisaccade percent error and latency and error rates in task switching.	Only male participants; small sample; limited information on medication regimen and testing protocol.	Oppositional defiant disorder, not specified the number of patients. One ADHD-I boy and one ADHD-C boy met the minimum criteria for generalized anxiety disorder (GAD).
**Caldani et al. 2019 [43]**	Case–control	21 ADHD vs. 21 control (sex- and age-matched)	~8	Two visual tasks, eye tracking, postural platform	Fixation	ADHD children had poor fixation capability and poor postural stability when compared to TD children.	No	Small sample; absence of simultaneous head–eye tracking; potential “ceiling effect” in dual-task condition; cross-sectional design limits causal inference.	No psychiatric comorbidities; dyslexia and neurological disorders excluded.
**Bucci et al. 2014 [44]**	Case–control	28 ADHD, 14 controls.	~9	Eye tracking, force platform	Fixation, pro and antisaccades and pursuits.	ADHD: children (off and on MPH) had pursuits and saccades (pro- and antisaccades) similar to controls, but showing more saccades during the fixation task.	Yes: 14 patients treated with MPH (formulation and dosage not specified).	Small sample size	Not reported
**Castellanos et al. 2000 [45]**	Case–control	32 ADHD females vs. 20 controls (females)	6–13	Eye tracking	Smooth pursuit, delayed response, go/no-go	ADHD: normal smooth pursuit; more errors and commissions in delayed response and go/no-go tasks.	No	Females only; potential learning effects	No Intellectual quotient < 80, evidence of medical or neurological disorders on examination or by history, Tourette’s disorder, or any psychiatric disorder requiring psychiatric treatment.
**Klein et al. 2002 [46]**	Case–control	27 ADHD males	10–15	Eye tracking	Pro-saccades and antisaccades	MPH reduces pro- and anti-saccadic reaction times, error correction times, and the proportion of direction errors during the anti-saccade task.	Yes: MPH Group 1 (*n* = 13) was first tested without MPH, and 1 week later with; Group 2 (*n* = 14) performed the reverse testing order.	Males only	Not reported
**Matsuo et al. 2015 [47]**	Case–control	37 ADHD (12 female and 25 male) and 88 controls	5–11	Eye tracking	Fixation, pro-saccades task in step and gap conditions to evaluate the gap effect, which is the difference in the reaction time between the two conditions	Children with ADHD had a significantly longer reaction time than controls (*p* < 0.01), and the gap effect was markedly attenuated (*p* < 0.01).	No	Not reported	Not reported

## Data Availability

Data is contained within the article.

## Abbreviations

The following abbreviations are used in this manuscript:

ADHD	Attention-Deficit/Hyperactivity Disorder
MPH	Methylphenidate
SLDs	Specific Learning Disorders
MGSs	Memory-Guided Saccades
VGS	Visually Guided Saccade
EOG	Electrooculography
EEG	Electroencephalography
